# Circulating levels of asprosin in children with obesity: a systematic review and meta-analysis

**DOI:** 10.1186/s12902-024-01565-w

**Published:** 2024-03-13

**Authors:** Yuwei Zhang, Yifei Zhang, Bao Yang, Simin Li, Ru Jia

**Affiliations:** 1 Key Laboratory of Shaanxi Province for Craniofacial Precision Medicine Research, College of Stomatology, Xi’an Jiaotong University, Xi’an, Shaanxi Province 710004 China; 2 Clinical Research Center of Shaanxi Province for Dental and Maxillofacial Diseases, Xi’an, Shaanxi Province 710004 China; 3 Department of Prosthodontics, College of Stomatology, Xi’an Jiaotong University, Xi’an, Shaanxi Province 710004 China; 4 Department of Hepatobiliary Surgery, The First Affiliated Hospital of Xi’an Jiaotong University, Xi’an, Shaanxi Province 710000 China; 5 Department of Gynecology and Obstetrics, The First Affiliated Hospital of Xi’an Jiaotong University, Xi’an, Shaanxi Province 710000 China; 6 Department of Digital Oral Implantology and Prosthodontics, College of Stomatology, Xi’an Jiaotong University, Xi’an, Shaanxi Province 710004 China

**Keywords:** Obesity, Asprosin, Adipokine, Children, Meta-analysis

## Abstract

**Background:**

Prior studies reported that elevated asprosin level was associated with obesity in adults and animal models. However, the relationship between asprosin level and children with obeisty remains controversial. The aim of our analysis was to systematically review available literatures linking asprosin and children with obesity for a comprehensive understanding of the relationship between circulating asprosin level and obesity in children.

**Methods:**

Eight databases were gleaned for studies published up to January 2024. Standard mean difference with 95% confidence interval (CI) and Fisher’s Z transformation was calculated to evaluate the relationship between asprosin level and children with obesity using the Review Manager 5.4 Software. Other indicators were measured via mean difference with 95% CI.

**Results:**

Six observational studies were included both in systematic review and meta-analysis. The current evidence indicated that no significant difference was observed in the level of circulating asprosin between the children with and without obesity (SMD = 0.37; 95% CI:—0.22–0.95, *p* = 0.22). However, Fisher’s Z transformation suggested the positive association of circulating asprosin levels and clinical index measuring the degree of obesity: total cholesterol (Fisher’s Z: 0.11, 95% CI: 0.02–0.20, *p* = 0.02).

**Conclusions:**

Circulating asprosin level was not independently related to childhood obesity currently. More rigorous longitudinal researches were required to disentangle the causations. However, the positive association of asprosin levels and total cholesterol indicated that asprosin might get involved in the lipid-metabolism of childhood obesity, asprosin might be a prospective bio-index and targeted treatment of total cholesterol metabolism besides the role of glucogenic and orexigenic.

**Trial registration:**

Prospero ID: CRD42023426476.

**Supplementary Information:**

The online version contains supplementary material available at 10.1186/s12902-024-01565-w.

## Background

The prevalence of childhood overweight and obesity in most westernized countries, and in developing countries as gradually emerged, has increased dramatically. Notably, it has become one of the main public health problems worldwide over the past four decades. Meanwhile, the impact of obesity could extend from childhood and adolescence well into adulthood, for children affected by obesity are more tend to undergo adulthood obesity with a rising risk of cardio-metabolic comorbid complications and co-occurring mental disorders [1–4].

Obesity is demonstrated by excessive accumulation of adipose tissue. And adipose tissue, not only be regarded as an essential energy storage organ, but also as an important endocrine organ, which secretes several metabolically-active molecules, including peptides, lipids, cytokines. Those substances contribute to the metabolism and energy homeostasis in muscles, liver, central nervous system and heart [5].

Among these substances, adipokines are expressly involved in the inflammatory response in the pathophysiological processes of morbid obesity, as the balance of anti- and pro-inflammatory adipokines has been impaired. All these trigger the inflammatory cascade and obesity-related cardio-metabolic complications, such as systemic insulin resistance (IR) and type 2 diabetes mellitus (T2DM) [6]. Wherein, IR as a major predisposition of impaired glucose tolerance, impaired fasting glucose and T2DM during childhood and adulthood; is known by the reduced glucose uptake of the whole body in response to physiological insulin levels [7–9].

Asprosin is a novel adipokine first described in 2016 through neonatal progeroid syndrome [10], which was found getting involved in the regulation of food intake, the maintaining of glucose and energy homeostasis. As a polypeptide and cleavage product of 140-amino acid long C-terminal encoded by exons 65 and 66 of Fibrillin 1 gene (FBN1) located in chromosome 15q21.1, asprosin is mainly secreted by adipocytes, for FBN1 is mainly expressed in mature adipose tissue, as well as other tissues, such as salivary glands; pancreatic β-cell and skin [11, 12]. Besides, asprosin activates the G protein-cAMP-PKA pathway when recruited to the liver, which propels the release of rapid glucose and insulin into the circulation. Meanwhile, regulated by the negative-feedback loop, glucose could suppress the level of asprosin thereof [13]. For the organism, the circulating level of asprosin is consistent with the hepatic glucose, which acutely rises during fasting and drops when refeeding, thus the circadian rhythmicity of asprosin level is coordinate to nutritional state. Pathologically elevated asprosin level could be found at mice and adults with obesity and/ or IR, and the related asprosin genetic deficiency could end up with low food intake and extreme leanness [13, 14]. Functions of glucogenic and orexigenic imply that asprosin could be a potential therapeutic target for the breakthrough of therapy towards pure obesity, T2DM and metabolic syndrome.

These findings in mice and adults suggest the indispensable role of asprosin in the pathogenesis of obesity, T2DM and metabolic syndrome. However, the clinal relationship between asprosin and children with or without obesity is still controversial. Previous researches have suggested either increased [15–18] or the opposing decreased [19, 20] asprosin levels in children with obesity as compared to those with normal weight. And either positive [15–18] or negative [19, 20] correlations between asprosin and several metabolic parameters are reported. Moreover, the potential association between asprosin and other cytokines remains to be explored.

So far, no related systematic review and meta-analyses about asprosin and the relationship with obesity are published. In order to systematically compare fasting circulating asprosin levels between obese and non-obese children in a larger population, and investigate the relationships of circulating asprosin with metabolic parameters and biochemical biomarkers to explore the pragmatic role of asprosin in the process of obesity in children, we performed a systematic review and series of meta-analyses aiming at the characteristics, overall levels, function and the potential correlation with obesity-related metabolic parameters of asprosin in childhood obesity.

## Methods

### Protocol and registration

This systematic review and meta-analysis were conducted in accordance with the preferred reporting items for systematic reviews and meta-analyses (PRISMA) guidelines [21] along with the Meta-analysis of Observational Studies in Epidemiology guidelines [22], and registered in the Prospective International Register of Systematic Reviews (PROSPERO, CRD42023426476) as “Asprosin level in children with obesity: a systematic review and meta-analysis” and updated it into “Circulating asprosin level in children with obesity: a systematic review and meta-analysis” after the full research of the literatures, with the approval from all authors.

### Focused question and search strategy

Our specific clinical focused question was constructed around the association between circulating asprosin level and children affected by obesity according to the PICO/PECO guidelines [23], specifically, “is there a difference in the circulating asprosin levels between children with obesity and those without obesity?”.

## PECO Question

(**P**atient) Children age under 19 years, with a specific statement of pre- pubertal /pubertal stage;

(**E**xposure) Obesity;

(**C**omparison) Normal weight as control;

(**O**utcome) Serum and/or plasma asprosin levels.

Thus, the corresponding systematic literature search was carried out from seven databases, including PubMed, the Cochrane Library, ScienceDirect, ISI Web of Knowledge, Scopus, Web of Science and OpenGrey, and limited the searches to the first 200-hit in Google Scholar. Furthermore, a manual search of the relevant literature on the reference lists of the included original studies was performed. Two reviewers, YW. Z. and YF. Z., screened clinal case-controlled studies addressing the research question in English from 2000 to January 2024. The search strategy included keywords related to ((obesity OR obese OR body mass index OR BMI-SDS) AND (children OR childhood OR pre-puberty OR puberty OR juvenile OR adolescence) AND (asprosin OR FBN1 OR Fibrillin 1 gene OR cytokine OR adipokine)) were summarized and any kind of disagreement between the two reviewers were resolved by a third author, R. J.

### Inclusion and exclusion criteria

Two reviewers (YW. Z. and YF. Z.) conducted the initial screening based on the titles and abstracts of the related articles, then full texts of the preliminary included articles and the controversial articles were carefully reviewed through the second screening. Discrepancies were resolved by a third investigator (R. J.) by consensus. To evaluate the chance-adjusted inter-rater agreement, Cohen’s κ- statistic was utilized and the κ-coefficient for this stage was 0.89, indicating substantial agreement.

Studies were considered eligible once they reached the following criteria: (1) the type of the study design was an observational study, including cross-sectional, case-controlled or prospective study. (Non-observational studies that describe baseline levels of asprosin can also be included for avoiding selection bias); (2) the study reported asprosin level of obese and non-obese children, and its association with children affected by obesity (whether with the severity of obesity, the clinical lipid metabolism indexes and so on); (3) obesity was the exposure and the control group were the children with normal weight (the obesity criterion for inclusion were listed in Table 1); (4) full-text were published in English; (5) the age of all participants ranged between 0—18 years old, participants did not have genetic and/or endocrine obesity; previous history of impaired glucose tolerance (IGT); use of medication or medical treatment with underlying metabolic effects; diabetes; chronic diseases; smoking. Studies were excluded once they were (1) in vitro or laboratory animal studies, case series or reports, review studies, conference abstracts, editor opinions, letters, expert comments; (2) available merely as abstracts or lacking of predefined outcome data required for analyses (despite efforts to retrieve raw values from the original authors).

**Table 1:** Characteristics of the included studies in the systematic review

Study	Design; setting	Obesity criterion for inclusion	Sample and Methods	Case group			Control group		
				Sample size	Average BMI (kg/m^2^) and age (yrs)	Biomarkers	Sample size	Average BMI (kg/m^2^) and age (yrs)	Biomarkers
Corica et al., 2021 [20]	Single-center, cross-sectional, case-controlled study; Italy	BMI ≥ + 2 SDS, in accordance with definition of obesity by WHO for children from the age of 5 years [24].	Serum (overnight fasting at least 8 h); ELISA	43 m/f: 21/22; Pre-p/p:17 /26	BMI ≥ + 2 SDS; Age: (11.9 ± 2.1) yrs	Asprosin:(331.9 ± 120.5) pg/mL	24 m/f: 7/17 Pre-p/p:10/14	BMI < + 2 SDS; Age: (10.7 ± 2.9) yrs	Asprosin:(358.1 ± 74.1) pg/mL
Long et al., 2019 [19]	Single-center, cross-sectional, case-controlled study; China	BMI greater than the age- and sex-appropriate 95th percentile based on the criteria of the BMI growth reference for Chinese children aged 0–18 years [25, 26].	Plasma (overnight fasting at least 12 h); ELISA	47 m/f: 26/21	Boys: BMI (24.77 ± 2.33) kg/m^2^; BMI SDS:(2.09 ± 0.47); Age: (11.04 ± 1.34) yrs.; Girls: BMI (20.80 ± 4.99) kg/m^2^; BMI SDS: (2.22 ± 1.08); Age: (8.29 ± 1.21) yrs	Asprosin:(9.24 ± 4.11) ng/mL	40 m/f: 23/17	Boys: BMI (16.17 ± 2.34) kg/m^2^; BMI SDS:(–0.52 ± 0.84); Age: (10.31 ± 1.76) yrs Girls: BMI (15.36 ± 1.61) kg/m^2^; BMI SDS: (–0.27 ± 0.95); Age: (8.91 ± 1.42) yrs	Asprosin:(12.33 ± 4.18) ng/mL
Wang et al., 2019 [15]	Single-center, cross-sectional, case-controlled study; China	BMI exceeded 95% of the normal level for a child of that age, according to the BMI reference criteria for Chinese children [27]. The BMI data were standardized to age-specific and sex-specific centiles based on the Working Group on Obesity in China Normal weight and were given as BMI SDS. WHR was calculated as waist (cm) divided by height (cm).	Serum (overnight fasting at least 12 h); ELISA	79 m/f: 56/23 Pre-p/p:38/41	BMI (27.44 ± 3.93) kg/m^2^; BMI SDS (2.88 ± 0.59); Age: (10.82 ± 2.12) yrs	Asprosin: 1.51 ± 0.44 ng/mL; TNF-α: 11.67 ± 5.31 pg/mL; Adiponectin:3.08 ± 1.41lg/mL; leptin: 71.89 (44.32–87.00) ng/mL	40 m/f: 20/20; Pre-p/p:21/19	BMI (15.67 ± 2.30) kg/m^2^; BMI SDS (–1.00 ± 1.02); Age: (10.94 ± 2.19) yrs	Asprosin: 0.96 ± 0.48 ng/mL; TNF-α: 3.97 ± 1.95 pg/mL; Adiponectin: 3.92 ± 1.74 lg/mL; Leptin: 7.07 (3.56–10.88) ng/mL
Silistre et al., 2020 [16]	Prospective study; Turkey	According to WHO Reference 2017 [24]: Low weight (BMI-SDS ≤ -1); normal weight (BMI-SDS >—1 and ≤ 1; overweight (BMI SDS > 1 and ≤ 2) and obesity (BMI-SDS > 2).	Serum (overnight fasting at least 12 h); ELISA	44 m/f: 20/24	BMI: 29.585(20.56) kg/m^2^; BMI SDS: 2.475(1.61); Age: (12.196 ± 2.62) yrs	Asprosin: 106.293 ± 122.69 ng/mL	60 m/f:32/28	BMI: 19.745 (7.82) kg/m^2^; BMI SDS: -.035(1.94); Age: (12.921 ± 2.51) yrs	Asprosin: 70.903 ± 17.49 ng/mL;
Liu et al., 2021 [17]	Single-center, cross-sectional, case-controlled study; China	According to the BMI reference for Chinese children, children with a BMI > 95% for their age and sex were diagnosed as having obesity. The BMI data were standardized to age- and sex-specific centiles and were converted into BMI SDS) [27].	Serum (overnight fasting at least 12 h); ELISA	75 m/f:52/26	BMI: (27.06 ± 3.24) kg/m^2^; BMI SDS: (3.514 ± 1.1); Age: (10.752 ± 2.02) yrs	Asprosin: 1.356 ± 0.419 μg/mL; TNF-α: 5.32 ± 4.90 pg/mL; Adiponectin:3.33 ± 1.50 μg/mL	35 m/f:19/16	BMI: (15.79 ± 2.28) kg/m^2^; BMI SDS: (− 0.53 ± 0.58); Age: (10.95 ± 2.29) yrs	Asprosin:1.12 ± 0.43 μg/mL; TNF-α: 1.81 ± 0.65 pg/mL; Adiponectin:3.85 ± 1.78 μg/mL
Moradi et al., 2023 [18]	Cross-sectional, case-controlled study; Iran	BMI higher than the 95th percentile related to age and gender entered the group with obesity.	Serum (overnight fasting 12–16 h); ELISA	35	BMI: (28.13 ± 5.75) kg/m2; Age: (132.57 ± 32.74) mo	Asprosin:8.65 ± 2.01 nmol/L; Visfatin: 2.09 ± 1.03 ng/mL; Metrnl: 72 ± 21.9 pg/mL	35	BMI: (18.41 ± 2.65) kg/m^2^; Age: (138.77 ± 25.65) mo	Asprosin:6.69 ± 1.97 nmol/L; Visfatin: 1.49 ± 0.87 ng/mL; Metrnl: 100 ± 27.84 pg/mL

*BMI* body mass index, *SDS* standard deviation score, *WHO* World Health Organization, *m/f* male/female, *Pre-p/p* pre-pubertal/ pubertal, *yrs* years, *mo* month, *WHR* Waist to-height ratio

### Data extraction and quality evaluation

The same two assessors (YW. Z. and YF. Z.) reviewed all of included literatures and the undertook data extraction independently based on the pivotal question addressed in this systematic review with the sole standardized data-collection form. Information was tabulated according to: the first author, publication year, designs; settings, obesity criteria, sample source, assay methods, characteristics of the case and control group, along with mean and standard deviation (SD) outcomes of the cytokines/adipocytokines investigated. The authors cross-checked all the extracted data, and any discrepancy was resolved by a discussion until consensus was reached. If a consensus could not be reached, a third experienced author (R. J.) was consulted. The κ-coefficient for inter-reviewer agreement was 0.92. The methodological quality of the selected observational-study was assessed according to a grading system developed by the Newcastle Ottawa Scale (NOS) [28]. As a validated evaluation instrument, NOS was recommended by the Agency for Healthcare Research and Quality and Methodological Index (AHRQ) for observational and non-randomized studies. The evaluation scale of NOS utilizes a star system consisting of three broad perspectives: the selection of the study groups on a scale of 0 to 4 stars; the comparability of the groups on a scale of 0 to 2 stars; and the ascertainment of the exposure/ outcome of interest on a scale of 0 to 3 stars, where form 0 to maximum stars indicates more adequate of report, lower risk of bias and higher level of quality. Notably, there are some few caveats on assessing the quality of our included studies that needed to be mentioned regarding to NOS, such as the missing items for the reliability, validity and appropriateness of the analysis, which acquired to be addressed and minimized. Firstly, confounding factors of each study need to be defined before giving the stars. Secondly, blinded is considered with higher score in quality assessment of case–control study in NOS, however, blinding exposure is sometimes impractical as the case–control status can be easily discerned, especially the obese status is accompanied by the apparent visual signs. It is more importantly to assess the interviews of each study were organized by highly standardized trained investigators.

### Statistical analysis

The original data extracted from the articles were presented as mean (M) ± standard deviation (SD). Standard mean difference (SMD) and the corresponding 95% confidence interval (95% CI) in the meta-analysis were calculated to estimate the differences in circulating asprosin levels between groups. Subgroup analyses were carried out to explore the potential source of heterogeneity considering race and gender. Moreover, the original data on homeostasis model assessment of insulin resistance (HOMA-IR), circulating levels of tumor necrosis factor-alpha (TNF-α) and adiponectin, which reflecting the biochemical features of obesity, were analyzed as mean difference (MD) and the 95% CI, since they got an approaching magnitude and consistent unit respectively. Besides, to further assess the association between circulating levels of asprosin and obesity in children, specifically, the association between asprosin and body mass index (BMI); BMI standard deviation score (BMI-SDS); HOMA-IR; age; total cholesterol (TC); triglycerides (TG); high-density lipoprotein cholesterol (HDL-C); low-density lipoprotein cholesterol (LDL-C); insulin; fasting blood glucose (FBG) and TNF-α, respectively, the Spearman or Pearson correlation coefficients of each study were recorded. Fisher’ Z transformation was used to estimate a mean transformed correlation weighted by each sample size given by the data in the included studies.

Significance levels of overall effects were determined by Z test, and forest plots were provided to demonstrate effect sizes and the corresponding 95% CI. Between-study heterogeneity was estimated by I^2^ statistic, which was based on the Cochran Q statistics [29, 30]. If I^2^ > 50%, substantial heterogeneity was assumed to exist and random effect model was used, otherwise, the fixed effect model was applied. Moreover, sensitivity analyses (leave-one-out analysis) were conducted by omitting one single study sequentially and calculating the influence on the pooled result of each study to test its robustness (by assessing whether the sensitivity analysis results are consistent with the main analysis results or not). Funnel plots were formed to assess publication bias in the meta-analyses, since the included studies of the subgroup analyses were limited, the potential publication bias with funnel plots couldn’t be evaluated. Even so, alternative methods or strategies are used to address publication bias as below: (1) The same positive research result repeatedly published in different journals must be removed. (2) Cultural background and political factors, which may affect the acceptance of results, if existed, must be considered. (3) The preferences of researchers, especially when they believe that research in a certain direction is of great significance, if existed, must be considered.

All statistical analyses were conducted via Review Manager (RevMan) 5.4 statistical software, which was provided by the Cochrane Collaboration. The significance *p* value was set as 0.05.

## Results

### Literature search

**Fig. 1:**
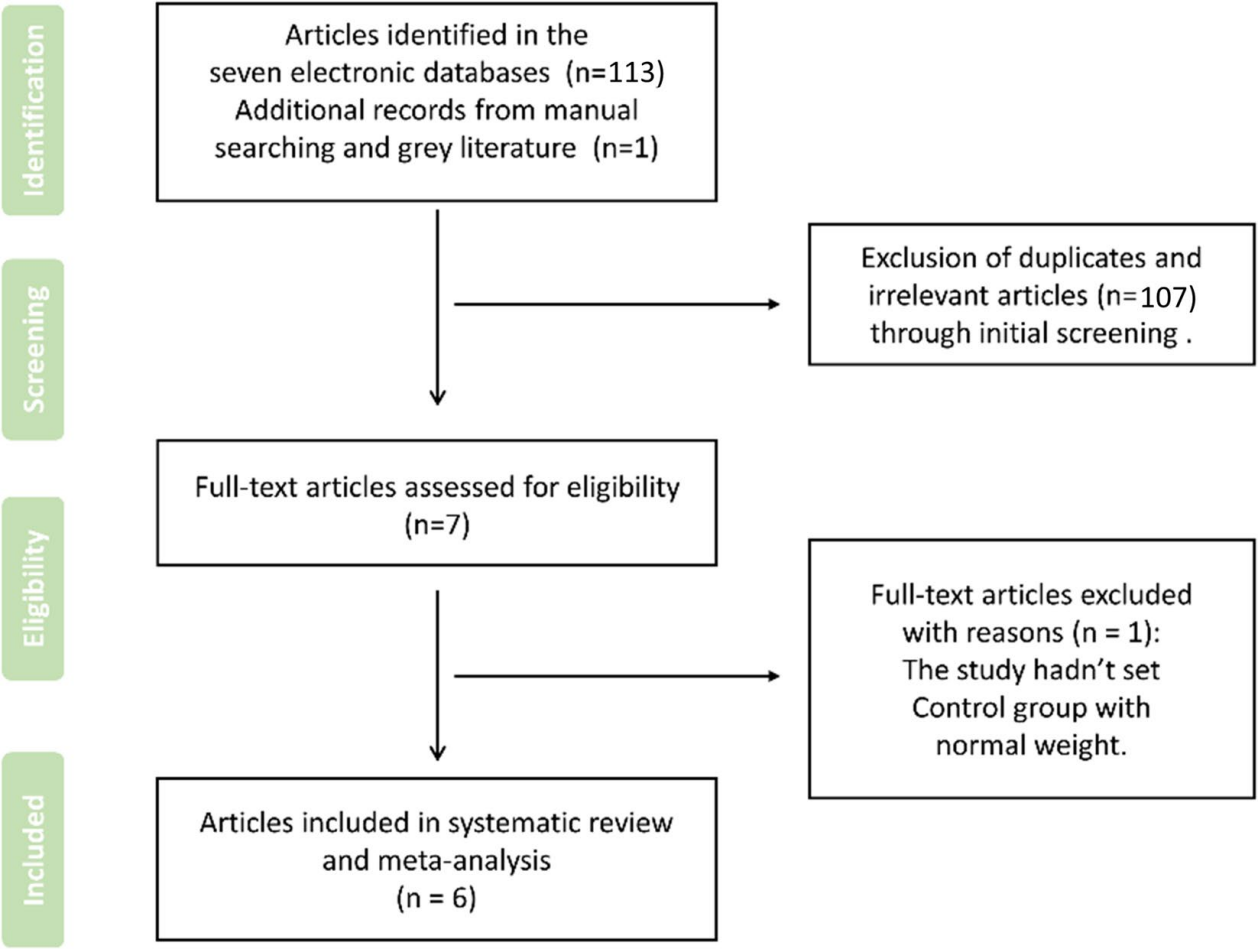
Flowchart presenting the search and the selection phases of the incorporated articles of the systematic review and meta-analysis

A total of 113 studies were yielded based on the search strategy from the electronic databases and 1 manuscript was identified from manual searching for consideration, no additional record was selected from gray literature. After initial screening of titles and abstracts, 107 studies which regarded as duplicates and irrelevant studies were removed from two reviewers independently. Then, the assessment of the full texts of the remaining 7 articles was proceeded, 1 study was deleted for unable to fulfill the inclusion criteria, and the specific reason was listed in Fig. 1. Thus, 6 studies were eventually considered eligible to be included in the present systematic review and meta-analysis. A flow diagram presenting all the aforementioned phases is displayed in Fig. 1.

### Characteristics and quality assessment of study

The characteristics of the included six studies published from 2019 to 2024 are depicted in Table 1. Of the six incorporated studies, half of them were set in China, Asia [15, 17, 19]; while the others were Caucasian, and were set in Turkey [16], Iran [18] and Italy [20] respectively, which reflected the diversity of ethnicities. Due to the study design of the selected population, though Turkey, Iran and China are all geographically located in Asia, the studies of the first two were classify as Caucasian in subgroup analysis. As for defining parameters of obesity in children, the World Health Organization (WHO) criteria [24] and the criteria of the BMI growth reference for Chinese children aged 0–18 years [25, 26] with or without assistant measures such as waist circumference, body fat, waist-hip ratio, and so on were taken into account. Expect for one study [19] was focusing on the fasting plasma level of aimed-biomarkers between groups through enzyme-linked immunosorbent assay (ELISA), others [15–18, 20] were measuring the fasting serum levels of biomarkers through ELISA. The sample size of these studies ranged from 67 to 158. The study performed by Corica et al., Long et al., Silistre et al., Liu et al. provided the data on male and female subjects separately considering gender as a probable confounding factor. Moreover, the Spearman or Pearson correlation coefficients of circulating asprosin level and obesity-related indexes were provided to investigate the potential correlations of asprosin with obesity-related parameters.

The assessment of the quality of the included observational studies using the Newcastle–Ottawa Scale was shown in Table 2. Scores ranged between 7–8 stars, indicating good quality.

**Table 2:** Quality assessment of incorporated studies using the Newcastle–Ottawa scale

Study	Selection	Comparability	Exposure	Score	Quality
Corica et al., 2021 [20]	★★★★	★	★★★	8	Good
Long et al., 2019 [19]	★★★★	★	★★★	8	Good
Wang et al., 2019 [15]	★★★★	★	★★★	8	Good
Silistre et al., 2020 [16]	★★★	★	★★★	7	Good
Liu et al., 2021 [17]	★★★★	★	★★★	8	Good
Moradi et al., 2023 [18]	★★★	★★	★★	7	Good

### Overall meta-analysis

**Fig. 2:**
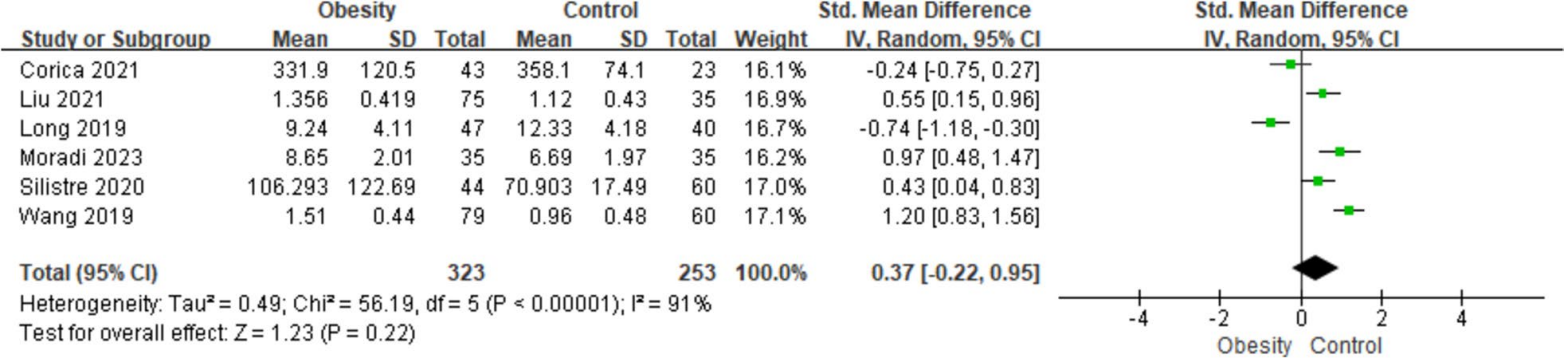
Forest plot of circulating asprosin level in children with obesity or normal weight. The random effect model and SMD with 95% CI were applied

As depicted in Fig. 2, the overall levels of circulating asprosin in children with obesity were not significantly different from that in the normal weight controls (SMD = 0.37; 95% CI:—0.22–0.95, *p* = 0.22). The SMDs from six incorporated studies were analyzed via the random-effects model, for the overall heterogeneity was considered significant (Tau^2^ = 0.49, Chi^2^ =56.19, I^2^ = 91%, *p* < 0.00001).

Despite of the limited included studies, no significant publication bias was found in funnel plot of the overall meta-analysis as displayed in Figure S1. Sensitivity analysis was further conducted in the combined results by removing one study in turn, while no dramatically relevant changes were showed, which suggesting that the current findings were robust against study removal.

### Subgroup analysis

#### Subgroup analysis based on geographic sites

**Fig. 3:**
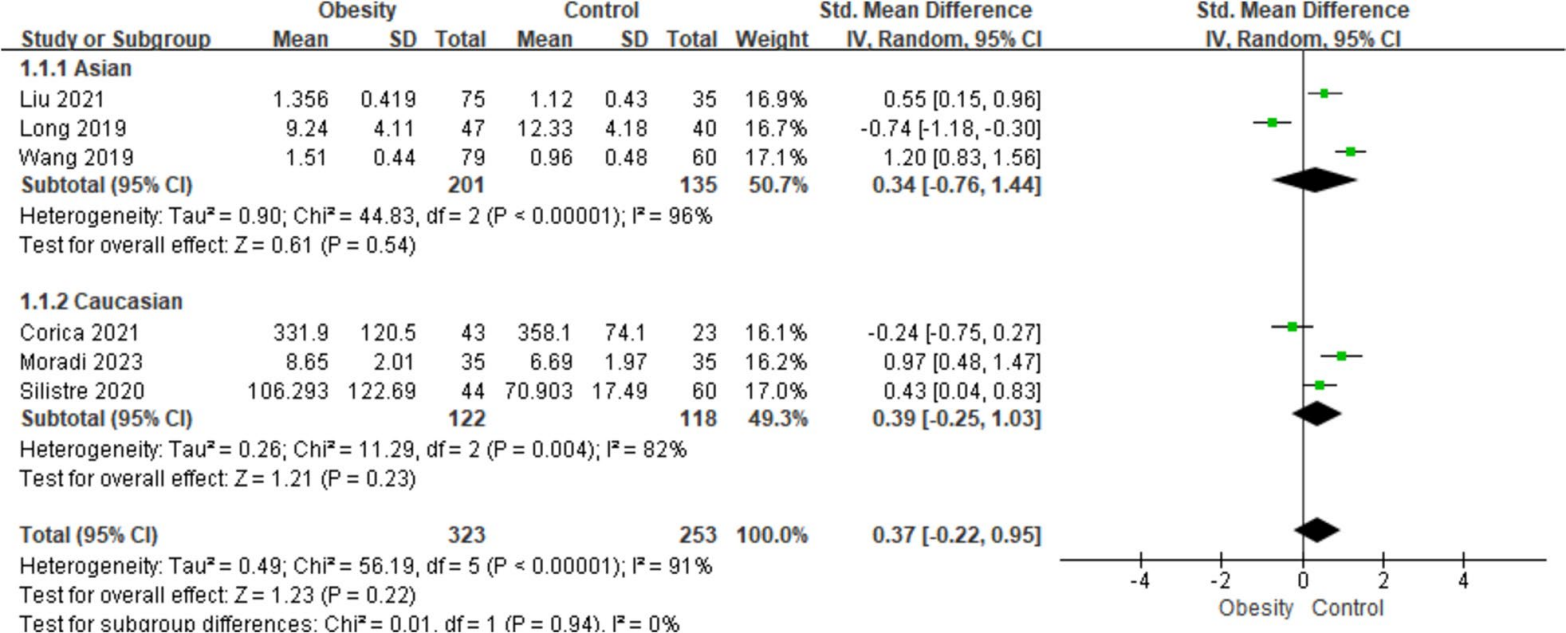
Subgroup analysis of asprosin levels in children with obesity or normal weight based on ethnic groups. The random effect model and SMD with 95% CI were applied

Further investigations about the potential source of heterogeneity and other essential information about the correlations between obesity-related indexes and circulating asprosin level were measured through subgroup analyses. Subgroup analysis was also first carried out based on ethnic groups/geographic sites (Fig. 3). When stratifying by ethnic groups, same amount of three studies were classified as Asian group and Caucasian group, and no significant difference was found in asprosin level between the participants with obesity and with normal weight (Asian group: SMD = 0.34; 95% CI: -0.76–1.44, *p* = 0.54; Caucasian group: SMD = 0.39; 95% CI: -0.25–1.03, *p* = 0.23).

#### Subgroup analysis based on gender

**Fig. 4:**
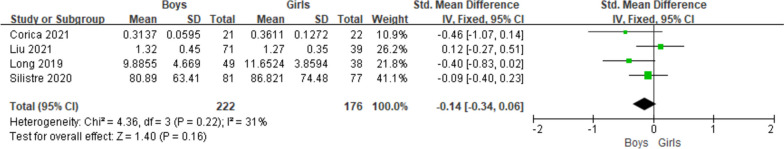
Subgroup analysis of asprosin levels in boys or girls based on gender. The fixed effect model and SMD with 95% CI were applied

Former studies suggest that gender might be a confounding factor in asprosin level [20], thus subgroup analysis was then conducted based on gender for the whole population as showed in Fig. 4. The fixed effect model was chosen since no significant heterogeneity was observed (I^2^ = 31%, *p* = 0.22), and the difference between boys and girls in respect to circulating asprosin level was not significant (SMD = -0.14; 95% CI: -0.34–0.06, *p* = 0.16).

#### Subgroup analysis based on HOMA-IR

Additionally, HOMA-IR is an essential indicator of insulin resistance. With regard to HOMA-IR values of the selected subjects, fixed effect model was chosen (I^2^ = 23%, *p* = 0.27) and subgroup analysis demonstrated that the value of subjects with obesity was significantly higher than control group (MD = 1.78; 95% CI: 1.47–2.08, *p* < 0.00001) as displayed in Figure S2. Meanwhile, concrete analysis was conducted and the subjects were divided into IR and non-IR with the random effect model (I^2^ = 90%, *p* = 0.002), the circulating asprosin level was no obvious difference between IR and non-IR participants (SMD = 0.63; 95% CI: -0.73–1.99, *p* = 0.37) as demonstrated in Figure S3. To further explore the association between asprosin and IR, Fisher’ Z transformation was chosen to calculate the given Spearman or Pearson correlation coefficient. Random effect models were used as significant heterogeneity was observed (HOMA-IR: I^2^ = 96%, *p* < 0.00001; insulin: I^2^ = 95%, *p* < 0.00001; FBG: I^2^ = 73%, *p* = 0.005). Due to the given data, results (Figures S4, S5 and S6) revealed no apparent association of asprosin with HOMA-IR; or of asprosin with insulin; or of asprosin with FBG, with a Fisher’s Z of 0.06 (HOMA-IR: 95% CI: -0.65 to 0.76, *p* = 0.88); 0.13 (insulin: 95% CI: -0.62 to 0.87, *p* = 0.74) and 0.08 (FBG: 95% CI: -0.10 to 0.26, *p* = 0.37).

#### Subgroup analysis based on BMI or BMI-SDS

**Fig. 5:**
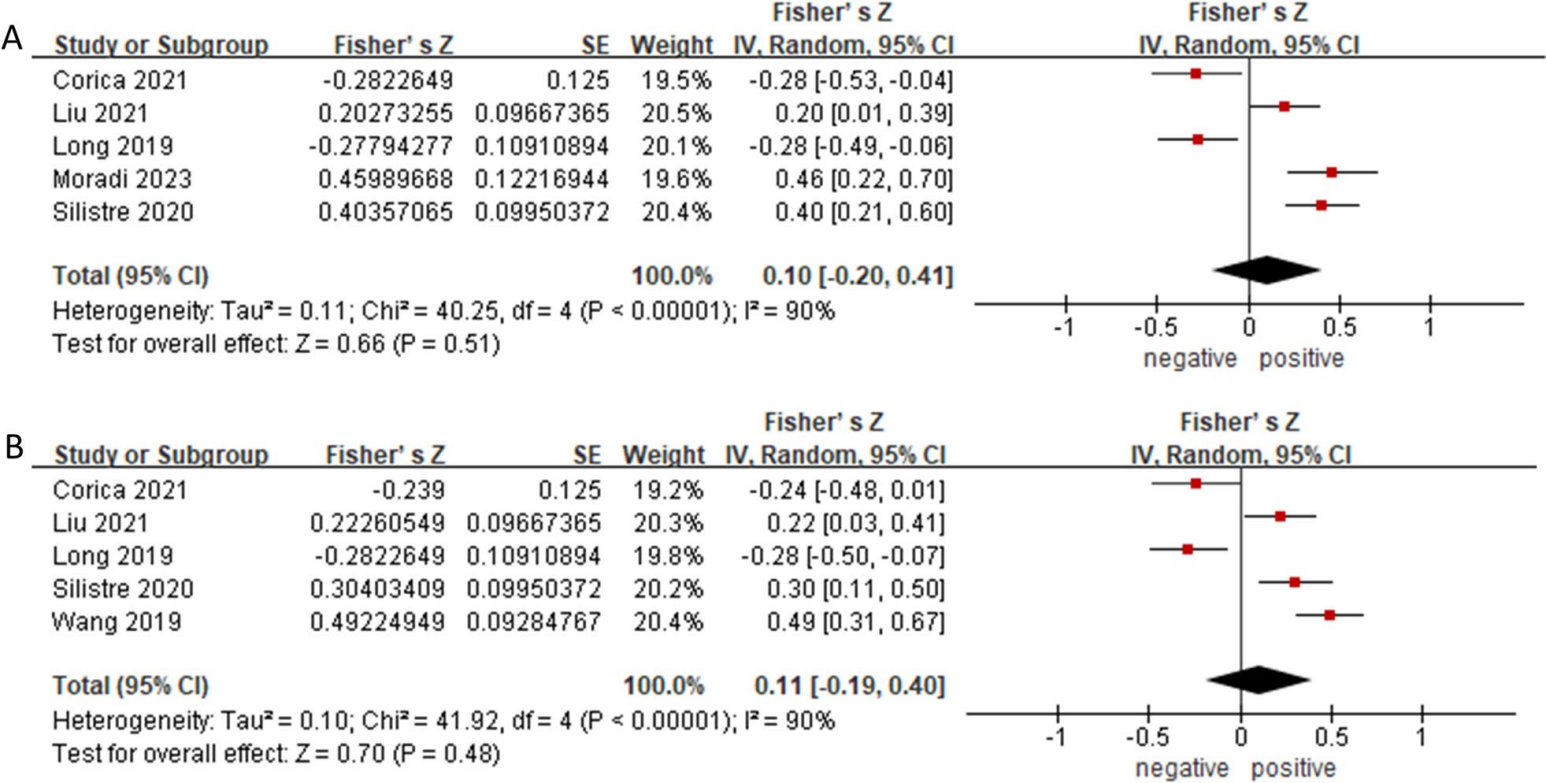
Subgroup analysis of the association of asprosin levels with BMI (**A**) and association of asprosin levels with BMI-SDS (**B**). The random effect model and Fisher’s Z with 95% CI were applied

As for the association between asprosin and BMI, significant heterogeneity was discovered (I^2^ = 90%, *p* < 0.00001) and the random effect model was chosen. Figure 5A showed no significant association of asprosin with BMI, neither negative or positive correlation. Fisher’s Z was 0.10 (95% CI: -0.20 to 0.41, *p* = 0.51). Similar situation occurred between asprosin and BMI-SDS (Fig. 5B), with Fisher’s Z of 0.11 (95% CI: -0.19 to 0.40, *p* = 0.70), which indicated that level of asprosin was not significantly associated with the severity of obesity when judging by BMI-related indexes And the results were robust against study deleting.

#### Subgroup analysis based on total cholesterol

**Fig. 6:**
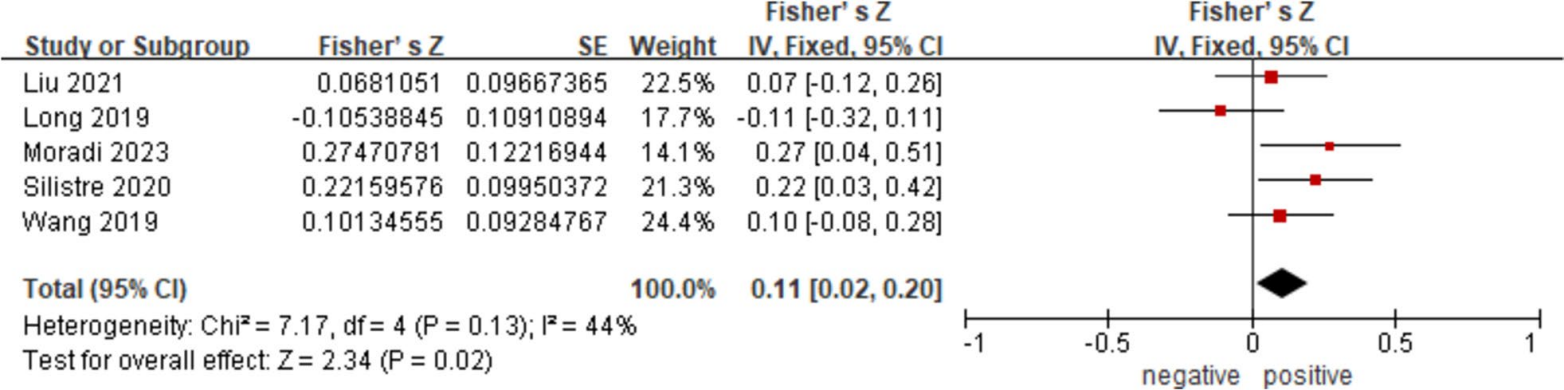
Subgroup analysis of the association of asprosin levels with TC. The fixed effect model and Fisher’s Z with 95% CI were applied

However, given by the data in five included observational studies of asprosin and TC, the result (Fig. 6) showed a rather significant positive correlation of asprosin and TC, with a Fisher’s Z of 0.11 (95% CI: 0.02 to 0.20, *p* = 0.02). No significant heterogeneity was observed (I^2^ = 44%, *p* = 0.13) and the fixed effect model was used for this analysis. This suggested a potential clinical correlation between total cholesterol metabolism and asprosin level. In more detail, when considering the association between asprosin and LDL-C or HDL-C (Figures S7 and S8), no significant correlation was found (LDL-C: Fisher’s Z of 0.02, 95% CI: -0.12 to 0.15, *p* = 0.79; HDL-C: Fisher’s Z of -0.02, 95% CI: -0.16 to 0.13, *p* = 0.83), neither in the association between asprosin and TG (Figure S9, Fisher’s Z of 0.12, 95% CI: -0.06 to 0.30, *p* = 0.20).

#### Subgroup analysis based on age

As Figure S10 suggested, no significant association between age and circulating asprosin level in this review was found (Fisher’s Z of -0.08, 95% CI: -0.18 to 0.02, *p* = 0.14).

#### Subgroup analysis based on TNF- α or adiponectin

Then we proceeded the subgroup analysis based on TNF- α or adiponectin, these anti- and pro-inflammatory indexes could assist in determining the level of inflammation in the body. Current data demonstrated a significant positive correlation of asprosin and TNF- α, as a pro-inflammatory indictor associated with obesity, with a Fisher’s Z of 0.39 (Figure S11, 95% CI: 0.07 to 0.71, *p* = 0.02). Besides, the levels of circulating TNF- α in children affected by obesity were significantly higher than that in the control group (Figure S12, MD = 5.59; 95% CI: 1.49–9.70, *p* = 0.008). However, the levels of circulating adiponectin, an anti- inflammatory indictor related to obesity, were significantly lower in children affected by obesity than that in the normal weight controls (Figure S13, MD = -0.70; 95% CI: -1.16–-0.24, *p* = 0.003).

## Discussion

Hitherto, the clinal relationship between asprosin and children with or without obesity is still controversial. Previous researches displayed either increased [15–18] or decreased [19, 20] asprosin levels in children affected by obesity compared to those with normal weight, and either positive [15–18] or negative [19, 20] correlations between asprosin and several metabolic parameters are reported. Hence, further investigation about the role of asprosin in the process of obesity in children, which is the topic of our meta-analysis, is needed. Our systematic review and meta-analysis revealed that, currently, the circulating asprosin levels in children with obesity were not significantly different from that in children with normal weight. Considering the overall heterogeneity, subgroup analyses were carried out in this review. Due to the inconsistent measurement methods or different types of utilized ELISA kits, and basically clinical heterogeneity, including samples, pre-analytical conditions, age, ethnicity, gender, physical conditions, etc., the actual detected asprosin levels varied from one to another, the combination of these confounding factors may result in the heterogeneity and explained why SMD in discrepancy situation was applied. As further subgroup analysis applied, ethnicity may not attribute to the heterogeneity. Either within Asian or Caucasian, the difference in levels of asprosin between groups remained insignificantly. Nevertheless, ethnicity is a rather complex factor, whether affect by the geographic locations, habits and customs and genetic differences, apparently given by the existing data is not quite enough.

Age, in some studies [15, 17–19] was proved to be negatively related to the circulating asprosin level. Nevertheless, as Figure S10 suggested, no significant association between age and circulating asprosin level in this review was found. The results without statistical differences might due to the masking of potential confounding factors, as aging is a process that accompanies various changes in the body, such as gender/gonadal hormones and nutrition.

Former studies suggested that gender may potentially influence the asprosin levels. Corica et al. discovered that asprosin was lower in boys than in girls while Long et al. propounded that asprosin was lower in boys than in girls with obesity [19, 20], and Wiecek et al. found that single anaerobic effort was more appealed to women in ascending asprosin compared to men [31]. Sex differences caused by gonadal hormones and sex chromosome complement in obesity, fat oxidation, thermogenesis, metabolic syndrome, lipid metabolism and other obesity-related complications are well established. Meanwhile, asprosin is mainly secreted from adipocyte and could regulate appetite [32, 33], it is a reasonable estimate that there might be gender difference in the association between asprosin and obesity-related indexes. However, the latest result in subgroup analysis of asprosin levels in boys or girls did not significantly support the different level of asprosin in different gender. This may be caused by the inconsistent and unstable hormone levels in different stages of children, such as pre-pubertal and pubertal stage; besides, the different degree of obesity may also lead to the results above.

As an essential index in metabolic diseases, HOMA-IR was taken into account to reflect the obesity status. Widely regarded that the patients with HOMA-IR values greater than 3.16 were recognized as having IR [34]. HOMA-IR values in groups with obesity in the included subjects were significantly greater than the control [14, 16, 18, 19], HOMA-IR mean values in three out of four studies were higher than 3.16 in groups with obesity, thus, most of the participants with obesity were suffered from insulin resistance. Notably, as a state of low-grade systemic inflammation (LGSI) [35], obesity could induce the dysfunction of adipose tissue and manifest as adipocyte hypertrophy, ectopic fat distribution and modifications in the cellular composition and intracellular matrix, and alter its endocrine function. All the risks mentioned above are attributed to development of IR in liver, skeletal muscle and other peripheral tissues [36]. Along with the mass increase in white adipose tissue, deep characteristic biochemical and histological changes in obesity-related inflammation appear. Growing evidence demonstrated that inflammation, as a pathogenic mediator is essential in the development of obesity-induced IR. What’s more, tissue-resident immune cells are generally accepted as the leading role in the obesity-induced inflammation [37]. The forementioned paracrine cycle between macrophages and adipocytes leads to the vicious cycle, expediting the adipose tissue inflammation subsequently. Via the actions in the hypothalamus, the systemically changed adipokine secretion level could lead to the increase in food intake, decline in energy consumption and insulin sensitivity of liver and muscle through enhancing inflammation and the accumulation of ectopic lipid [38–40]. Consequently, the disruptions of adipokine secretion result in the formation and deterioration of obesity and IR. Even recent genetic researches proved that several bio-molecules are involved in the development of obesity-induced inflammation and IR [39], the actual function of asprosin is still obscure. Given by the scarce and contradictory date displayed either in asprosin levels in IR or non-IR groups or the association of asprosin levels with HOMA-IR in the included subjects, we could not conclude that asprosin is significantly related to IR. The correlation of HOMA-IR or insulin and asprosin levels were neither negative nor positive, mainly depended on the decreased or increased asprosin levels in subjects with obesity stated in each study. The adipose tissue and liver would strive to maintain metabolic homeostasis by secreting adipokines under normal physiological conditions.

However, the homeostasis could be disrupted in obesity status, which results in the increasing level of asprosin in adults and mice with obesity and IR and further accelerates the release of hepatic glycogen while aggravates IR [41]. The current mainstream view is that asprosin level and IR were positively correlated, regardless of the IR in T2DM, metabolic syndrome, non-alcoholic fatty liver disease or comorbidities [15, 42–46]. However, the effect of asprosin is complex in the real world, due to the varied medical history of patients and thus the level of asprosin is not necessarily merely associated with or affected by IR. One study demonstrated that asprosin level did not increase as a prevention or compensation towards hypo glycaemia. This may not only relate to IR, but also most probably as an early stage of NAFLD with the alterations in the structure of liver [47]. This is a process involving a complex feedback mechanism, which wherein the causality cannot be made simply by observational studies. Also, intriguingly, asprosin is wildly known for its glucogenic function, while in our meta-analysis the association of asprosin levels with fasting blood glucose is not significant. All this might be contributed to the particularity of childhood. First, according to the given mean HOMA-IR values, not all participants with obesity were suffered from IR. Besides, the difference in the BMI of participants is another confounding factor. Due to the dose-dependent relationship between adverse health outcomes and BMI, children under a relatively metabolically healthy “honeymoon phase” [48] could maintain in the compensatory period of metabolic balance through the reducing secretion of asprosin. This is also the reasonable explanation for why the level of asprosin may be positively correlated to BMI or BMI-SDS when exceeding one cut-off point of relatively high BMI or BMI-SDS. However, as regarded to the entire cohort, this positive correlation relationship was not stable, which suggested stratified BMI/BMI-SDS evaluation based on the characteristics of childhood obesity for correlation between asprosin level and BMI/ BMI-SDS should be considered.

Furthermore, the positive association of asprosin levels with TC was found to be significant. Widely known that childhood obesity elevates total cholesterol and blood pressure [49], the association of asprosin levels with TC may recommend another clinical sensitive parameter and underlying pathway in understanding the function of asprosin in the metabolism of total cholesterol. Compared to adults, there is greater heterogeneity in childhood asprosin level, which may be due to the changes in age.

During the process of chronic LGSI, the adipose tissue expands and releases chemokines. These chemokines then adhere to the endothelial cells and gradually migrate and leak within the adipose tissue to trigger the infiltration of macrophages and inflammation [50]. Then the macrophages activate the generation of pro-inflammatory cytokines, notably as interleukin 6 (IL-6), IL-1β and TNF-α through cross-talking with the parenchymal adipocytes [51], meanwhile the level of anti-inflammatory cytokines such as IL-10 and adiponectin begin to decrease. Our results are consistent with those findings, which indicated the positive association of asprosin levels with TNF-α levels, and TNF-α levels in groups with obesity were significantly higher than the control in the included subjects. The opposite consequent occurred when adiponectin levels in groups with obesity were significantly lower. Macrophages, play an essential role in secreting TNF-α and other major inflammatory stoking source in adipose tissue, and all these macrophage-derived factors attribute and deteriorate to LGSI [52]. Taking TNF-α as an example, through inducing the release of saturated fatty acids by lipolysis, TNF-α could lead to inflammatory-related changes in macrophages via Toll-like receptor 4. Meanwhile, the generation of adiponectin, leptin, visfatin, retinol binding protein-4 and resistin become irregularly and the production of free fatty acids begins to increase, then the obesity-related syndromes occurred and worsen [53]. As aforementioned statement, based on the analysis of existing data about the correlations between circulating asprosin and various parameters, obese children might have higher level of HOMA-IR and circulating TNF-α, as well as the lower level of circulating adiponectin than non-obese children. These clinical indexes might reflect the severity of the obesity. Especially, when the biochemical analysis of circulating asprosin is above the average value of that in children with normal weight, it might suggest the total cholesterol in blood is also of high value and worth noting.

In summary, our systematic review and meta-analysis conducted a relatively thorough and comprehensive analysis about the circulating level of asprosin in childhood obesity and their relationship. Asprosin is a novel adipokine first described in 2016, and our proposal provided the latest evidence and filled in the vacancies of systematic review and meta-analysis about the relationship between asprosin and obesity in human, particularly in the special population of children. It cannot be neglected that the long-term effects and the related adverse outcomes on obesity during the childhood, and we therefore propounded more convincing details about the interaction of asprosin and obesity.

However, novelty inevitably accompanies scarcity, given by the limited data and mostly included cross-sectional studies, there are some issues that acquires further improvements (i.e., explore on the cause-effect relationship) and in-depth understanding. First, the cause of the overall heterogeneity had been stated above, however, the factor of physical conditions of subjects could be more extensive which led to the conflicting results from each study and reduce the reliability. Though participants with medication history had been excluded, whether patients exercise (such as climbing the stairs or rushing into the detection spot and etc.) before the blood was taken could also affect the asprosin level. Evidence had accumulated as the level of asprosin can decrease after acute aerobic exercise both in the morning and evening, what’ s more, the level varied from different timepoint after the exercise [54]. Also, the fasting time, circadian rhythm and the degree of obesity could also alter the asprosin level. Second, the initial aim of the present study was to analyze the relevance from a public health perspective and speculate the underlying pathophysiological pathways by accumulating statistical assessments. Yet our current comparisons were mainly based on cross-sectional studies of the observational studies, it was inadequate to investigate the cause–effect relationship between asprosin level and childhood obesity. Therefore, more coming prospective studies, along with in vitro and in vivo experimental studies or intervention studies should be included to clarify the actual effect of how asprosin act in the process of childhood obesity. Third, given by rather limited studies and sample size of each,, any results should be interpreted scrupulously. And publication bias was unavoidable, since articles without English publication or incomplete data had not been included.

Due to the insufficient sample size and studies, types of design, ethical and other reasons in the real world, potential mechanisms are not clarified clearly. Apparently further related studies should be carried out to investigate the role of asprosin in childhood obesity, provide the circulating biomarker reference values and discover the targeted treatment of obesity. It is more rational to set stratification based on stage (pre-pubertal/ pubertal); sample (plasma/ serum); obesity comorbid with other disease or not; the degree of obesity; ethnic group; gender; measurement methods. Strict quality control must be applied in the consistent physical conditions of the subjects before analysis, well-designed prospective cohort study or intervention study and blinding (at least in the laboratory testing section) in the analytical process.

All in all, further studies should focus on sufficient sample size, strict stratification in regards to confounding factors, well-designed study and strict quality control. Expectance on the deep and therapeutic investigation of asprosin in childhood obesity is meaningful and worthwhile.

## Conclusions

To sum up, our systematic review and meta-analysis of included high-quality observational studies revealed that circulating level of asprosin in children with obesity or without obesity was of no statistical difference, which suggesting circulating asprosin level was currently not independently related to childhood obesity. Rigorous longitudinal researches controlled for the related confounding factors were required to disentangle the exact causations between asprosin and children affected by obesity. However, childhood obesity was known to be associated with elevated total cholesterol, and we found the positive association of asprosin levels and total cholesterol, which indicated that asprosin might participate in the development of lipid-metabolism of childhood obesity. Asprosin might be a prospective bio-index and targeted treatment of total cholesterol metabolism besides the role of glucogenic and orexigenic.

### Supplementary Information

**Supplementary Material 1.**

## Data Availability

The datasets used and/or analyzed during the study are available from the corresponding author upon reasonable request.

## Abbreviations

IR: Insulin resistance
T2DM: Type 2 diabetes mellitus
FBN1: Fibrillin 1 gene
PRISMA: Preferred reporting items for systematic reviews and meta-analyses
NOS: Newcastle Ottawa Scale
AHRQ: The Agency for Healthcare Research and Quality and Methodological Index
M: Mean
SD: Standard deviation
SMD: Standard mean difference
95% CI: 95% Confidence interval
HOMA-IR: Homeostasis model assessment of insulin resistance
TNF-α: Tumor necrosis factor-alpha
MD: Mean difference
BMI: Body mass index
BMI-SDS: BMI standard deviation score
TC: Total cholesterol
TG: Triglycerides
HDL-C: High-density lipoprotein cholesterol
LDL-C: Low-density lipoprotein cholesterol
FBG: Fasting blood glucose
RevMan: Review Manager
WHO): World Health Organization
ELISA: Enzyme-linked immunosorbent assay
m/f: Male/female
Pre-p/p: Pre-pubertal/ pubertal
yrs: Years
mo: Month
WHR: Waist to-height ratio
LGSI: Low-grade systemic inflammation
IL-6: Interleukin 6
