# Supplementary material for: Circulating levels of asprosin in children with obesity: a systematic review and meta-analysis
Source: BMC Endocr Disord. 2024 Mar 13;24:36. doi: 10.1186/s12902-024-01565-w (PMC10936088; doi:10.1186/s12902-024-01565-w)
Supplement: Supplementary file 1 — Supplementary Material 1. [file 12902_2024_1565_MOESM1_ESM.docx]

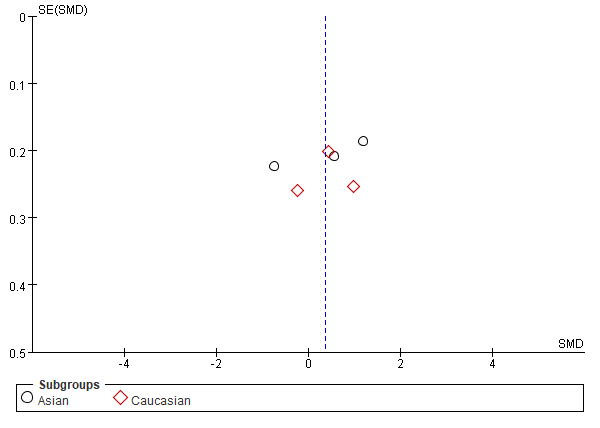


**Figure S1.** Funnel plot of included six studies for potential publication bias according to ethnic groups.


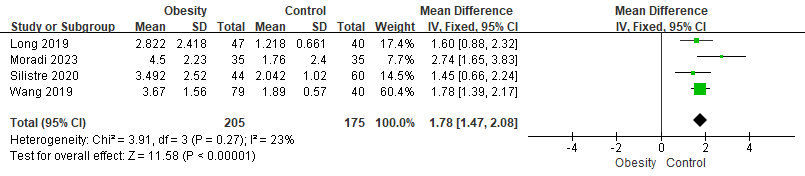


**Figure S2.** Subgroup analysis of HOMA-IR values in groups with obesity or normal weight in the included subjects. The fixed effect model and MD with 95% CI were applied.


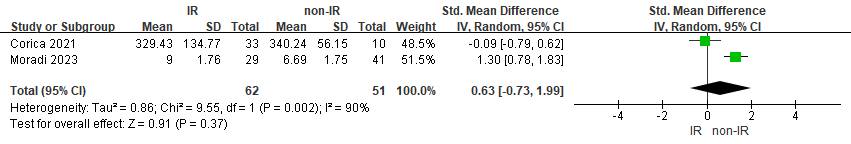


**Figure S3.** Subgroup analysis of asprosin levels in IR or non-IR groups in the included subjects. The random effect model and SMD with 95% CI were applied.


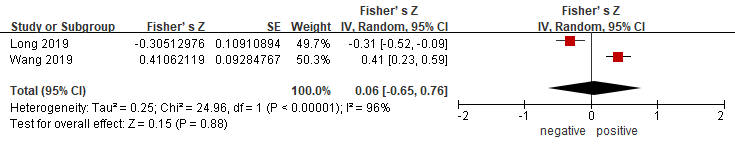


**Figure S4.** Subgroup analysis of the association of asprosin levels with HOMA-IR. The random effect model and Fisher’s Z with 95% CI were applied.


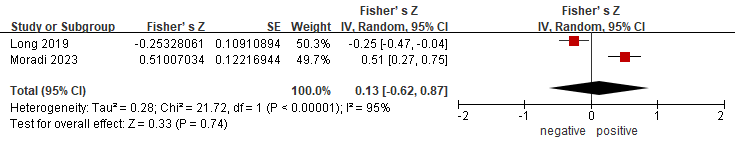


**Figure S5.** Subgroup analysis of the association of asprosin levels with insulin levels. The random effect model and Fisher’s Z with 95% CI were applied.


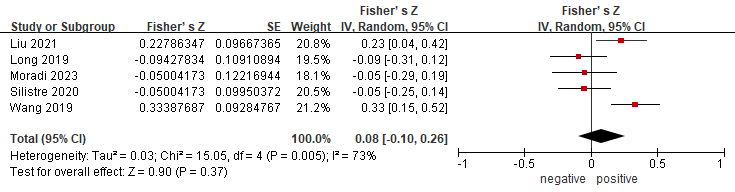


**Figure S6.** Subgroup analysis of the association of asprosin levels with FBG. The random effect model and Fisher’s Z with 95% CI were applied.


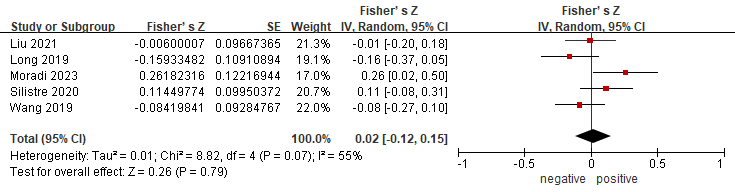


**Figure S7.** Subgroup analysis of the association of asprosin levels with LDL-C. The random effect model and Fisher’s Z with 95% CI were applied.


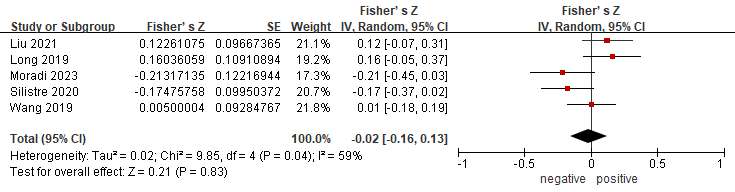


**Figure S8.** Subgroup analysis of the association of asprosin levels with HDL-C. The random effect model and Fisher’s Z with 95% CI were applied.


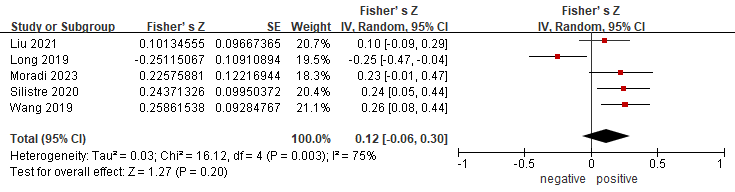


**Figure S9.** Subgroup analysis of the association of asprosin levels with TG. The random effect model and Fisher’s Z with 95% CI were applied.


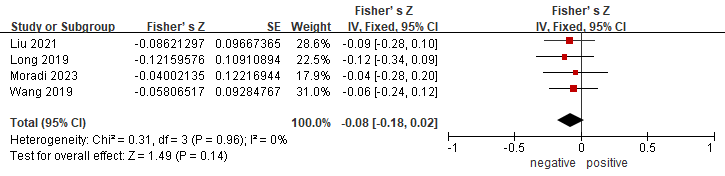


**Figure S10.** Subgroup analysis of the association of asprosin levels with age. The fixed effect model and Fisher’s Z with 95% CI were applied.


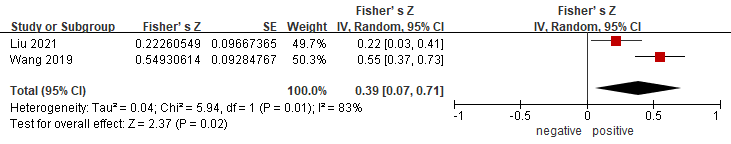


**Figure S11.** Subgroup analysis of the association of asprosin levels with TNF-α levels. The random effect model and Fisher’s Z with 95% CI were applied.


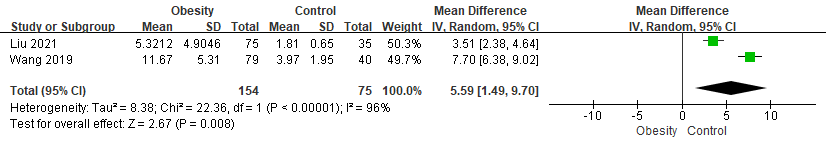


**Figure S12.** Subgroup analysis of TNF-α levels in groups with obesity or normal weight in the included subjects. The random effect model and MD with 95% CI were applied.


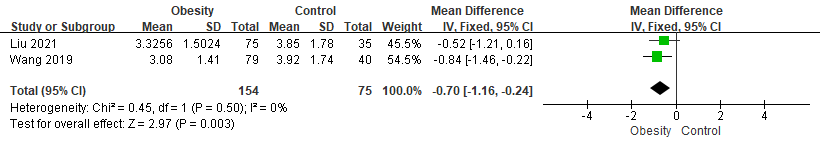


**Figure S13.** Subgroup analysis of adiponectin levels in groups with obesity or normal weight in the included subjects. The fixed effect model and MD with 95% CI were applied.
